# Endocarditis After Redo TAVR Managed With Medical Treatment and Later TAVR-in-TAVR-in-TAVR

**DOI:** 10.1016/j.jaccas.2025.105652

**Published:** 2025-10-22

**Authors:** Pedro Henrique Ferro de Brito, André Moreira Nicolau, Pedro Sérgio Soares Jallad, Marco Antônio Smiderle Gelain, Thiago Abizaid Kleinsorge, Marco Antônio Perin, Silvia Maria Pinella Helaehil, João Cavalcante, Alexandre Antônio da Cunha Abizaid, Fábio Sândoli de Brito Júnior

**Affiliations:** a Instituto do Coração da Faculdade de Medicina da Universidade de São Paulo (InCor/HCFMUSP), São Paulo, Brazil; b Hospital Sírio Libanês, São Paulo, Brazil; c Allina Health Minneapolis Heart Institute, Minneapolis, Minnesota, USA

**Keywords:** infective endocarditis, redo TAVR, structural valve degeneration, transcatheter aortic valve replacement (TAVR), valve-in-valve

## Abstract

**Background:**

Infective endocarditis after transcatheter aortic valve replacement (TAVR) is a rare but serious condition, with high morbidity and mortality, often requiring surgery.

**Case Summary:**

An 89-year-old woman with prior TAVRs (Sapien 3 and Evolut Pro) presented with dyspnea and fever. Blood cultures grew *Staphylococcus hominis*, and imaging confirmed endocarditis with minimal aortic regurgitation. She was deemed inoperable owing to age and comorbidities and was treated with lifelong antibiotics. After 18 months, she developed severe aortic regurgitation and heart failure due to structural valve degeneration (SVD). Active infection was excluded, and TAVR-in-TAVR-in-TAVR using a 20-mm Sapien 3 Ultra was performed successfully.

**Discussion:**

This case illustrates the feasibility of prolonged medical management for post-TAVR endocarditis in high-risk patients, with redo TAVR for subsequent SVD.

**Take-Home Message:**

Lifelong antibiotics and redo TAVRs in case of later SVD may be viable in frail patients, avoiding the risks of surgery.

## Graphical Abstract

**Figure:**
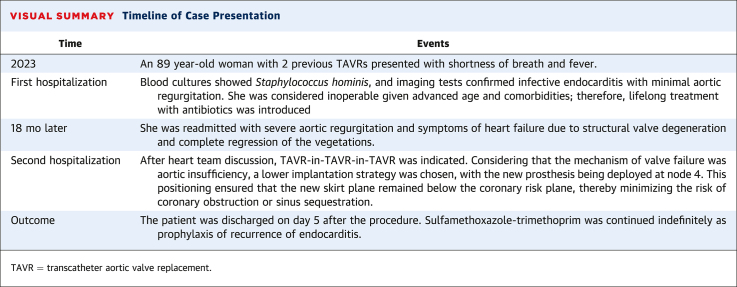

## History of Presentation

**Figure 1:**
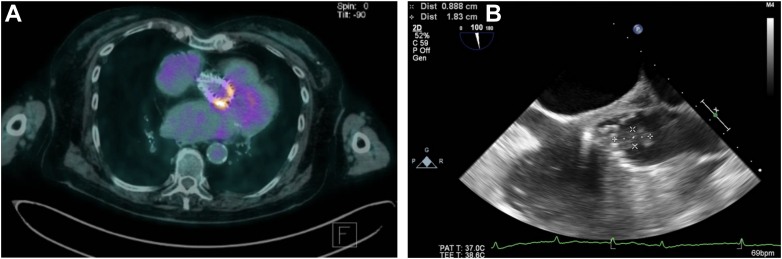
Imaging findings at Initial Presentation (A) PET-CT with enhanced metabolic expression in the proximal and medial area of the transcatheter valve (SUV_max_: 6.4). (B) Transesophageal echocardiogram with thickened leaflets and presence of an irregular, cotton-like mobile mass in the arterial side of the endoprosthesis leaflets, extending into the aortic root, measuring 1.8 cm in length and 0.9 cm in width. PET-CT = positron emission tomography–computed tomography; SUV_max_ = maximum standardized uptake value.

An 89-year-old woman presented to the emergency department after 5 days of dyspnea and fever. Initial work-up demonstrated elevated inflammatory markers; blood cultures showed *Staphylococcus hominis* in all 3 samples taken. Transesophageal echocardiogram, positron emission tomography–computed tomography, and computed tomography angiography (CTA) were consistent with the diagnosis of bacterial endocarditis with bulky vegetations (Figure 1, Video 1) and minimal aortic regurgitation. Initial medical treatment included 6 weeks of rifampicin, ertapenem, and teicoplanin, followed by sulfamethoxazole-trimethoprim after discharge for prevention of recurrence. After 18 months of clinical management, the patient was readmitted owing to worsening dyspnea and edema. Initial physical examination suggested significant aortic regurgitation.

**Take-Home Messages**

• Lifelong antibiotic therapy is an unconventional but possible alternative for frail or high–surgical risk patients with post-TAVR endocarditis, given the high morbidity and mortality rates of TAVR explantation.
• Redo TAVRs might be needed during the follow-up as a lifesaving procedure for SVD that may occur earlier in this situation.

## Past Medical History

The patient had undergone transcatheter aortic valve replacement (TAVR) with a 23-mm Sapien 3 for degenerative aortic stenosis in 2017 and redo TAVR with a 23-mm Evolut Pro in 2021 owing to structural valve degeneration (SVD) of the transcatheter heart valve. Other significant cardiovascular comorbidities included hypertension and atrial fibrillation, for which she was on long-term anticoagulation with apixaban.

## Investigations

**Figure 2:**
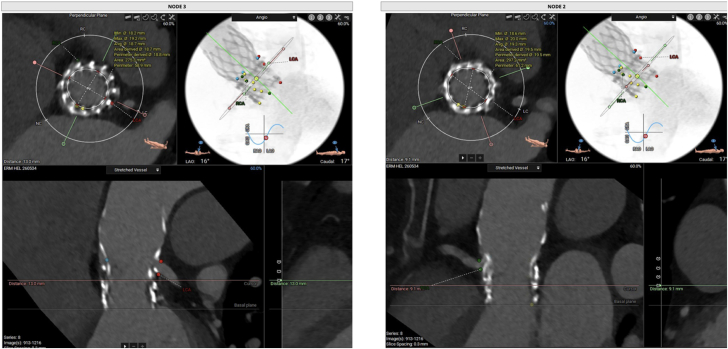
Computed Tomography Angiography-Based Measurements on the 23-mm Evolut Pro Nodes 2 and 3 The measurements demonstrated an area of 297.2 mm^2^ for node 2 and 275.3 mm^2^ for node 3.

New transthoracic echocardiogram demonstrated severe aortic regurgitation (Video 2), CTA showed complete regression of the vegetations (Figure 2), and new positron emission tomography–computed tomography demonstrated resolution of the inflammatory process.

## Management

**Figure 3:**
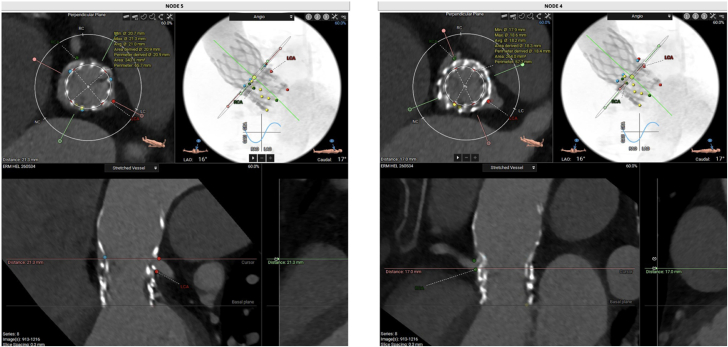
Computed Tomography Angiography-Based Measurements Including Estimation of Potential New Skirt Planes at Nodes 4 and 5 and Area Measurement The new skirt plane at node 4 was 21.3 mm and was chosen for safe deployment below the coronary risk plane. The area at node 4 was 264 mm^2^. CTA = computed tomography angiography.

After a heart team discussion, TAVR-in-TAVR-in-TAVR was indicated. The CTA was analyzed for procedure planning using 3mensio software (3mensio Medical Imaging) (Figures 2 and 3). Valve sizing was based on the average of the annular areas measured at levels corresponding to nodes 2, 3, and 4 (297.2, 275.3, and 264 mm^2^, respectively). The coronary risk plane was located between nodes 4 and 5. Therefore, considering that the mechanism of valve failure was aortic insufficiency, a lower implantation strategy was chosen, with the new prosthesis being deployed at node 4. This positioning ensured that the new skirt plane remained below the coronary risk plane, thereby minimizing the risk of coronary obstruction or sinus sequestration. A TAVR-in-TAVR-in-TAVR was performed using a 20-mm Sapien 3 Ultra (Edwards Lifesciences), followed by postdilation with an 18-mm Atlas Gold balloon (Becton Dickinson) at 18 atm (Videos 3 and 4) to optimize prothesis expansion and reduce the risk of patient-prothesis mismatch, which was of particular concern given the small nominal valve diameter. Final echocardiogram demonstrated excellent hemodynamics, with no paravalvular leaks and a mean gradient of 6 mm Hg (Video 5).

## Outcome and Follow-Up

The patient was discharged on day 5 after the procedure. Sulfamethoxazole-trimethoprim was continued indefinitely as prophylaxis for recurrence of endocarditis. At the 1 year follow-up, the patient was asymptomatic, with a mean gradient of 7 mm Hg.

## Discussion

Infective endocarditis is an uncommon but serious complication after TAVR, with an incidence ranging from 1% to 3% at 1 to 2 years postprocedure and 3.25% at longer follow-up durations.^1^, ^2^, ^3^ Outcomes are generally poor, with high rates of stroke (5%-16%) and mortality (1-year mortality ranging from 26% to 38%; 5-year mortality up to 60%)^2^, ^3^, ^4^, ^5^, ^6^ irrespective of treatment strategy. Regarding microbiology, *Enterococcus* species are the most frequently isolated pathogens, followed by *Staphylococcus aureus* and the coagulase-negative species found in the present case.^2^ Whether the incidence of infective endocarditis is higher after TAVR-in-TAVR is yet to be determined. Surgical treatment with explantation of the transcatheter heart valve, followed by surgical aortic valve replacement, is needed in approximately 20% of the patients and carries a high risk of morbidity and mortality.^7^, ^8^, ^9^ This case illustrates the successful management of infective endocarditis after a redo TAVR in a nonagenarian patient treated medically, followed by TAVR-in-TAVR-in-TAVR after an early SVD. It is probable that the endocarditis, although controlled by the medical treatment, contributed to the early SVD of the transcatheter heart valve, causing fragility and rupture of its leaflets.

## Conclusions

This case highlights the complexities of managing infective endocarditis in patients after TAVR and highlights a nonconventional management of this life-threatening disease for inoperable or high–surgical risk patients, using prolonged antibiotic therapy and redo TAVRs in case of subsequent SVD. Rigorous procedure planning for redo TAVRs is key to its success.

## Funding Support and Author Disclosures

The authors have reported that they have no relationships relevant to the contents of this paper to disclose.

## Appendix

Video 1 Filamentous Hypoattenuating Image Adhered to the Left Aortic Valve Leaflet, Measuring Approximately 2.4 × 1.5 × 0.8 cm, With an Appearance That May Be Related to a Bulky Vegetation and/or Thrombus Significant diffuse hypoattenuating leaflet thickening (HALT) was demonstrated and was estimated to involve more than 75% of the leaflets in the posterior and left coronary sinuses.
